# Investigating subtypes of lung adenocarcinoma by oxidative stress and immunotherapy related genes

**DOI:** 10.1038/s41598-023-47659-8

**Published:** 2023-11-27

**Authors:** Guangliang Duan, Changxin Huang, Jiangang Zhao, Yinghong Zhang, Wenbin Zhao, Huiping Dai

**Affiliations:** 1 Department of Oncology, Hangzhou Normal University, Affiliated Hospital, Hangzhou, 310015 Zhejiang China; 2 Department of Oncology, Shaoxing Cent Hospital, Shaoxing, 312030 Zhejiang China; 3 Department of Nephrol, Hangzhou Normal University, Affiliated Hospital, Hangzhou, 310015 Zhejiang China; 4 Hangzhou Normal University Affiliated Hospital, Hangzhou, 310015 Zhejiang China; 5 Department of Proctol, Hangzhou Normal University, Affiliated Hospital, Hangzhou, 310015 Zhejiang China

**Keywords:** Cancer, Drug discovery

## Abstract

Lung adenocarcinoma (LUAD) is one of the most widespread and fatal types of lung cancer. Oxidative stress, resulting from an imbalance in the production and accumulation of reactive oxygen species (ROS), is considered a promising therapeutic target for cancer treatment. Currently, immune checkpoint blockade (ICB) therapy is being explored as a potentially effective treatment for early-stage LUAD. In this research, we aim to identify distinct subtypes of LUAD patients by investigating genes associated with oxidative stress and immunotherapy. Additionally, we aim to propose subtype-specific therapeutic strategies. We conducted a thorough search of the Gene Expression Omnibus (GEO) datasets. From this search, we pinpointed datasets that contained both expression data and survival information. We selected genes associated with oxidative stress and immunotherapy using keyword searches on GeneCards. We then combined expression data of LUAD samples from both The Cancer Genome Atlas (TCGA) and 11 GEO datasets, forming a unified dataset. This dataset was subsequently divided into two subsets, Dataset_Training and Dataset_Testing, using a random bifurcation method, with each subset containing 50% of the data. We applied consensus clustering (CC) analysis to identify distinct LUAD subtypes within the Dataset_Training. Molecular variances associated with oxidative stress levels, the tumor microenvironment (TME), and immune checkpoint genes (ICGs) were then investigated among these subtypes. Employing feature selection combined with machine learning techniques, we constructed models that achieved the highest accuracy levels. We validated the identified subtypes and models from Dataset_Training using Dataset_Testing. A hub gene with the highest importance values in the machine learning model was identified. We then utilized virtual screening to discover potential compounds targeting this hub gene. In the unified dataset, we integrated 2,154 LUAD samples from TCGA-LUAD and 11 GEO datasets. We specifically selected 1,311 genes associated with immune and oxidative stress processes. The expression data of these genes were then employed for subtype identification through CC analysis. Within Dataset_Training, two distinct subtypes emerged, each marked by different levels of immune and oxidative stress pathway values. Consequently, we named these as the OX^+^ and IM^+^ subtypes. Notably, the OX^+^ subtype showed increased oxidative stress levels, correlating with a worse prognosis than the IM^+^ subtype. Conversely, the IM^+^ subtype demonstrated enhanced levels of immune pathways, immune cells, and ICGs compared to the OX^+^ subtype. We reconfirmed these findings in Dataset_Testing. Through gene selection, we identified an optimal combination of 12 genes for predicting LUAD subtypes: ACP1, AURKA, BIRC5, CYC1, GSTP1, HSPD1, HSPE1, MDH2, MRPL13, NDUFS1, SNRPD1, and SORD. Out of the four machine learning models we tested, the support vector machine (SVM) stood out, achieving the highest area under the curve (AUC) of 0.86 and an accuracy of 0.78 on Dataset_Testing. We focused on HSPE1, which was designated as the hub gene due to its paramount importance in the SVM model, and computed the docking structures for four compounds: ZINC3978005 (Dihydroergotamine), ZINC52955754 (Ergotamine), ZINC150588351 (Elbasvir), and ZINC242548690 (Digoxin). Our study identified two subtypes of LUAD patients based on oxidative stress and immunotherapy-related genes. Our findings provided subtype-specific therapeutic strategies.

## Introduction

In 2020, lung cancer accounted for the highest number of cancer deaths, with approximately 2.2 million new cases and 1.8 million deaths^1^. Lung cancer can be classified into two broad categories: small-cell lung cancer (SCLC, 10–15%) and non-small-cell lung cancer (NSCLC, 80–85%)^2^. NSCLC predominantly consists of lung adenocarcinoma (LUAD), which represents 60% of all cases^3^. Major risk factors for LUAD include cigarette smoking, environmental contaminants, genetic factors, and alcohol consumption^4^. Unfortunately, LUAD is frequently diagnosed at advanced stages, resulting in a poor prognosis^5^. Furthermore, despite treatment with surgery and chemotherapy, patients diagnosed with LUAD often experience relapses and metastases^6^. The average 5-year survival rate for patients with LUAD is less than 20%^7^. Therefore, the identification of promising treatment targets and compounds for LUAD is crucial.

The correlation between oxidative stress and cancer cells is firmly established, as cancer cells exhibit significantly higher levels of oxidative stress in comparison to normal cells^8^. The elevated baseline level of reactive oxygen species (ROS) is a reflection of persistent oxidative stress generated by increased metabolism and abnormal cell development. For example, moderately elevated oxidative stress has been shown to promote tumor growth by encouraging cell transformation^9^, proliferation^10^, and survival^11^. However, the impact of oxidative stress can be either pro-tumor or anti-tumor, depending on the context^8^. Higher levels of oxidative stress can potentially induce the death of cancer cells. In LUAD therapy, several tyrosine kinase inhibitors (TKIs) have been utilized to induce cancer cell apoptosis via oxidative stress^12^. Owing to the biphasic impact of oxidative stress on tumorigenesis, further investigations are imperative to unravel its intricate mechanisms.

The interaction between PD-L1 and PD-1 signaling is a potent mechanism for inhibiting T cell activation, making it a promising therapeutic target^13^. Immune checkpoint blockade (ICB) has emerged as a critical treatment option for various diseases, including metastatic melanoma, NSCLC, and bladder cancer. Research suggests that ICB treatment may also impact oxidative stress, and the modification of oxidative stress may enhance the efficacy of ICB treatment^14^. Given the substantial heterogeneity of LUAD, individuals with the same clinical stage of LUAD may have different prognoses when treated with ICB. Therefore, categorizing LUAD based on sequencing data is crucial for personalized and effective LUAD therapy.

In recent years, there has been a widespread use of gene expression analysis to identify molecular subtypes, as evidenced in several publications. For instance, a study used immunogenomic profiling of 29 immune signatures to identify three distinct LUAD subtypes (Immunity High, Immunity Medium, and Immunity Low)^15^. Similarly, Qin et al. utilized critical immune-prognosis genes for patients with LUAD and stratified the patients into low-immunity and high-immunity subtypes^16^. In another study, researchers performed consensus clustering on differentially expressed aging-related genes in the Cancer Genome Atlas (TCGA) database. This analysis identified three clusters of TCGA-LUAD patients with substantial differences in prognosis, immune infiltration, chemotherapy, and targeted therapy^17^. However, more studies are needed to investigate the molecular subtypes of LUAD.

In this investigation, we combine 12 datasets with different sources into one single dataset and then separate it into the Dataset_Training and Dataset_Testing. We identified molecular subtypes of LUAD based on genes associated with oxidative stress and the immune system in the Dataset_Training. Furthermore, we compared the differences in prognosis, oxidative stress, and immune checkpoint genes between two molecular subtypes. We constructed four machine learning models to predict the subtype of LUAD by 12 genes which were selected by differentially expressed Genes (DEG) analysis and gene selection. These results, including subtypes and models were validated in the Dataset_Testing.

## Materials and methods

### Data collection, pre-processing and dividing

TCGA (The Cancer Genome Atlas) and GEO (Gene Expression Omnibus) are among the most frequently accessed databases for researchers working on various cancer types, including LUAD. In our research, we extracted RNA-seq data normalized by Transcripts Per Million (TPM), together with the associated clinical information from the TCGA-LUAD cohort, using the 'TCGAbiolinks' R package. In our exploration of the GEO database, we conducted a comprehensive search using specific keywords (detailed in Supplementary Table 1). We then filtered the datasets based on the following criteria: (1) The datasets should contain LUAD samples. (2) The expression data should represent mRNA expression from bulk LUAD samples. (3) There should be more than 12,000 genes in the expression data. (4) Essential overall survival details, such as survival status and time, must be available. (5) Each dataset should comprise more than 50 samples. (6) Datasets must be free of duplicate samples within the GEO database. To access standardized mRNA expression data and clinical information from the sorted datasets, we utilized the "GEOquery" package^18^. It's important to highlight that only LUAD samples equipped with both mRNA expression data and survival particulars were retained for subsequent analysis.

Given that the datasets we analyzed originate from diverse databases, platforms, and research groups, addressing the batch effect is crucial. In molecular biology, batch effects arise when non-biological variables in an experiment introduce variations in the generated data. If not accounted for, these effects can lead to misleading conclusions, especially when the factors causing the batch effects correlate with experiment outcomes. To mitigate these batch effects, we employed a binary transformation method. For instance, in a specific GEO dataset, if the CD8A gene expression ranges from 0 to 1000 with a median value of 500, our method transforms the CD8A gene expression of each LUAD sample into either 0 or 1—depending on whether the expression is above or below the 500 threshold. This method was applied to all genes and all datasets in our study. Subsequently, we integrated all the samples from both TCGA and GEO datasets, resulting in a unified dataset named 'Dataset_All'. Samples from 'Dataset_All' were randomly and equally partitioned into 'Dataset_Training' (50%) and 'Dataset_Testing' (50%).

### Oxidative stress and immune-related genes (OSIRGs)

To identify OSIRGs, we searched the GeneCards database using the keywords "oxidative stress" and "immunotherapy"^19^. The search results provide relevance scores that reflect the significance of genes in relation to "oxidative stress" and "immunotherapy". For instance, when considering "oxidative stress," these scores are determined by analyzing the co-occurrence of genes with "oxidative stress" within Medline documents. A higher number of co-occurrences, relative to what would be expected by chance, results in a higher relevance score (https://www.genecards.org/Guide/GeneCard). This score signifies the degree of association, with higher values indicating a stronger relationship of the gene to oxidative stress. It's important to note that the absolute values of relevance score lack significance and only provide the relative importance, with the order holding significance. Therefore, it's not advisable to set a fixed threshold (like a score of 10) for both "oxidative stress" and "immunotherapy." Specifically, we filtered and identified the top 1000 genes that displayed the highest relevance scores for both "oxidative stress" and "immunotherapy". After combining these selected genes, we ended up with a set of 2,000 genes. However, after accounting for overlaps, 1757 unique genes were identified. Among these 1757 genes, 1311 genes have available expression data in 'Dataset_Training' and 'Dataset_Testing. These genes, which we termed Oxidative Stress and Immunotherapy Relevant Genes (OSIRGs), were the foundation for our subsequent analyses.

### Acquisition of LUAD subtypes and gene set enrichment analysis (GSEA)

It’s important to note that the expression values of OSIRGs in Dataset_Training and Dataset_Testing were either 0 or 1.To cluster the LUAD samples in the Dataset_Training and Dataset_Testing, we employed the consensus clustering (CC) analysis. CC is a resampling-based technique developed to evaluate the stability of clusters uncovered within a dataset^20^. Through iterative sub-sampling of the dataset and subsequent clustering of these subsets, CC seeks to establish whether the same samples consistently form clusters together. The CC procedure encompasses several steps: multiple random samplings of data subsets, application of a clustering algorithm to each subset, and comparative analysis of clustering outcomes across all subsets to generate a consensus or average result. The CancerSubtypes package was utilized for conducting the CC analysis with same parameters in the 'Dataset_Training' and 'Dataset_Testing', individually and independently^21^. In our analytical framework, several critical parameters were meticulously set to ensure accuracy and reliability. Firstly, we designated the number of repetitions (reps) as 100, facilitating ample iterations for the analysis. The clustering algorithm was selected as “Partitioning Around Medoids” (PAM). PAM shares similarities with the K-means clustering approach, it uniquely employs medoids from the dataset as opposed to centroids^22^. This characteristic enhances the robustness of PAM, making it more resilient to noise and potential outliers. Finally, as a measure of distance, we incorporated the Pearson correlation coefficient, denoted as distance = "pearson", to compute the relationships within our data. Following the acquisition of subtypes through CC analysis, we employed the Kaplan–Meier (KM) curve method to evaluate survival differences across the identified subtypes.

Then, we employed the differentially expressed genes (DEG) from 'edgeR' R package to calculate the log_2_FoldChange (FC) and adjusted p-values of genes between LUAD tumor subtypes^23^. This procedure was applied to the expression data of both 'Dataset_Training' and 'Dataset_Testing', where the gene expression values were either 0 or 1. Subsequently, pathway enrichment analyses were performed on the log2FC and p-values of genes using the 'fgsea' R package^24^, in conjunction with the gene sets file (h.all.v2023.1.Hs.symbols.gmt). Hallmark gene sets, sourced from the MSigDB resource^25^, encapsulate specific, well-defined biological states or processes, and consistently demonstrate coherent expression patterns.

### Differential analysis of oxidative stress, tumor microenvironment (TME), and immune checkpoint genes among tumor subtypes

The gene set variation analysis (GSVA) algorithm offers a means to transform an mRNA expression matrix into another matrix of specific gene sets^26^. Essentially, this algorithm computes the enrichment scores of given gene sets for each sample. Our study used two distinct oxidative stress gene sets. The first, GOBP_Positive_Regulation_Of_Response_To_Oxidative_Stress, comprises 10 genes that either activate or amplify the response to oxidative stress. The second, Hallmark_Reactive_Oxygen_Species_Pathway, contains 49 genes known to be upregulated by reactive oxygen species (ROS). To assess the differences in these oxidative stress gene sets across subtypes, we applied the Wilcox test for statistical significance. This analysis was consistently executed on both the Dataset_Training and Dataset_Testing, adhering to identical parameters.

We employed the "estimate" package to calculate the immune, stromal, and tumor scores, which are the main components of TME^27^. To estimate the absolute abundance of eight different cell types, which included fibroblasts, endothelial cells, and immune cells, we utilized the MCPcounter technique based on the transcriptome data^28^. This process, maintained with consistent parameters, was applied to both Dataset_Training and Dataset_Testing, and the resultant differences in TME cells between subtypes were visually represented through heatmaps.

In both the Dataset_Training and Dataset_Testing datasets, we had available gene expression values for key immune checkpoint genes, specifically for PD1 (PDCD1) and CTLA-4 (CTLA4). We subsequently extracted the expression levels of these immune checkpoint genes for further analysis. To statistically evaluate the variations in the expression of these genes across subtypes, we employed the Wilcoxon test. Notably, unlike TME cells, the expression values of PD1 and CTLA-4 in both datasets had undergone binary transformation, resulting in values of either 0 or 1, which makes it hard to visualize the expression values by boxplot. Consequently, we made subtype comparisons of PD1/CTLA-4 expressions within each distinct dataset and used the expression data before the binary transformation, including TCGA-LUAD and the 11 GEO datasets, individually.

### Feature selection, model construction, and model validation

In the Dataset_Training, OSIRGs with a log_2_FC greater than 0.5 and an adjusted p-value of less than 0.05 were selected for model construction to predict subtypes. Subsequently, our study adopted gene selection techniques based on the Support Vector Machine (SVM) method using Recursive Feature Elimination (RFE) as proposed in a published article^29^. In the SVM-RFE algorithm, the ranking coefficient is constructed based on the weight vector derived from the SVM during its training process^30^. With each iteration, the algorithm discards the gene with the lowest ranking coefficient, thereby ensuring that genes with marginal importance or redundancy were filtered out. The goal was to identify optimal combinations of 3, 6, 9, 12, 15, and 18 genes through the SVM-RFE method. To validate the reliability of our chosen genes, we performed a fivefold cross-validation. The choice of the best gene combination was based on the point where the accuracy value reached stability. Here's a brief overview of the SVM-RFE process: (1) The SVM-RFE algorithm ranks genes based on a weight vector obtained from the SVM during its training phase. (2) In each iteration, the gene with the least ranking coefficient, indicating the least importance, is eliminated. (3) Using SVM-RFE, we sought optimal combinations of 3, 6, 9, 12, 15, and 18 genes. (4) To ensure the robustness of our selected genes, we applied a fivefold cross-validation. The optimal gene combination was determined at the point where the accuracy value plateaued or stabilized.

We utilized various methods for developing a predictive model to distinguish between OX^+^ and IM^+^ subtypes. These methods encompassed Decision Tree (DT), Support Vector Machine (SVM), Artificial Neural Networks (ANN), and Random Forest (RF), all of which were implemented using the caret package in R. The process of parameter tuning was conducted on the Dataset_Training by metrics like mean accuracy and mean AUC. To counteract the risk of overfitting, we employed 50 replicates of fivefold cross-validation to estimate Accuracy and AUC. For Decision Tree, we focused on the complexity parameter (cp), a parameter influencing model complexity and subsequently impacting model accuracy. For ANN, the "size" parameter dictated the number of units or nodes in the hidden layer, while the "decay" parameter acted as a regularization parameter to counter overfitting. In the context of RF, the "mtry" parameter held significant importance as it determined the count of randomly selected variables (or features) during each tree split. In the case of SVM, two pivotal parameters were sigma and C. The parameter "sigma" influenced the kernel's spread or width, while "C" was a regularization parameter controlling the trade-off between margin maximization and classification error minimization. Through a grid search, we explored a range of potential parameter values to identify the combination that yielded optimal performance of mean AUC and accuracy on Dataset_Training. Subsequently, for external validation, we applied the developed models to the Dataset_Testing to discern between the OX^+^ and IM^+^ subtypes. This evaluation again relied on AUC and Accuracy as performance metrics. Furthermore, we quantified the importance values of genes integrated into the selected model, and the gene with the highest importance value was selected as the hub gene.

### Survival analysis and potential compounds for hub gene

In the Dataset_Training and Dataset_Testing, we used survival R package to calculate the difference of high and low of expression values of genes. And the survival plots were plotted by the survminer R package. In this study, we employed virtual screening and molecular docking approaches to validate the association between the selected hub gene and compounds. To obtain the protein structure, we retrieved it from the AlphaFold database^31^. The Zinc15 database was used to retrieve the three-dimensional structures of compounds that have been approved by the food and drug administration (FDA)^32^. Then, AutoDock Vina was employed for virtual screening, pinpointing compounds with the lowest binding energy indicative of stable binding^33^. Binding energy is determined by evaluating the sum of interaction energies, like hydrogen bonds, and then subtracting the energy required to destabilize the initial structures. Within AutoDock Vina, this binding energy parameter helps determine the ligand with the most stable interaction with the protein. A more negative binding energy signifies a stronger and more favorable drug-target interaction. The top 4 small molecules with the lowest binding energy were selected, and their binding complex with the protein were visualized using Pymol software.

## Results

### Datasets searching and sorting

**Figure 1:**
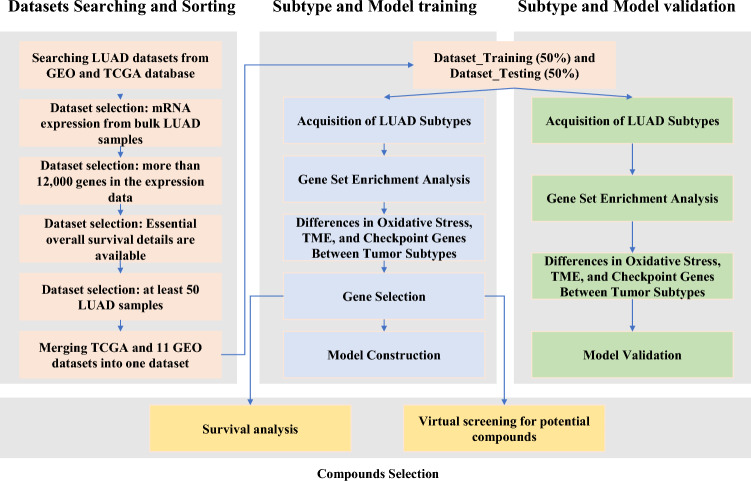
The flowchart of this study.

**Figure 2:**
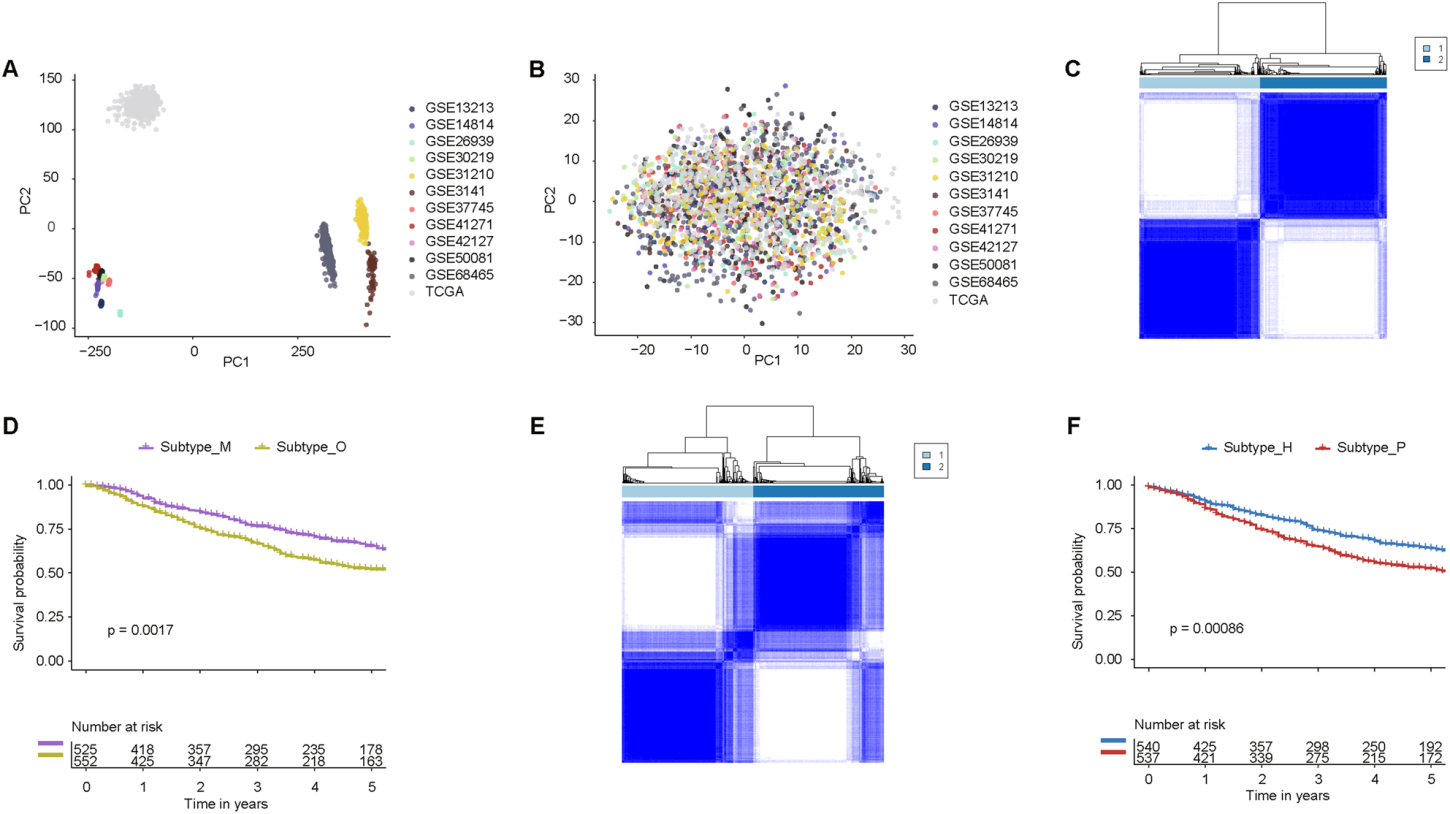
Identification of LUAD subtypes through consensus clustering (CC) using OSIRGs. (**A**) Principal component analysis (PCA) of 12 datasets prior to batch effect correction. (**B**) PCA of the 12 datasets following batch effect correction. (**C**) Heatmap of the consensus matrices for two subtypes in the Dataset_Training. Values range from 0 (never grouped together) to 1 (always clustered together), denoted by a color gradient from white to blue. (**D**) Overall Survival (OS) curves for subtypes with two subtypes in Dataset_Training. (**E**) Heatmap representing consensus matrices for two subtypes in the Dataset_Testing. (**F**) OS curves for subtypes within the two subtypes in Dataset_Testing.

The methodology flow of our study is depicted in Fig. 1. Initiating with our search strategy (outlined in Supplementary Table 1), we initially retrieved 269 datasets from GEO database. Upon employing our predefined sorting criteria, this was refined down to 11 suitable GEO datasets for subsequent analysis. Comprehensive clinical details, encompassing attributes like gender, TNM stage, age, survival status, and survival time for both the chosen GEO datasets and TCGA-LUAD, are delineated in Table 1. To address the inherent batch effects across these datasets, we adopted a binary transformation technique. An in-depth explanation of this methodology is provided in the “Methods” Section. To counteract the inherent batch effects observed across the datasets, we applied a binary transformation approach. A comprehensive description of this methodology is elaborated upon in the “Methods” Section. We utilized Principal Component Analysis (PCA) plots for a visual representation of the batch effect. Prior to the binary transformation, a pronounced batch effect was evident, as shown in Fig. 2A. Post transformation, this effect was significantly mitigated, as depicted in Fig. 2B. The number of RNAs with available expression value in each dataset are different, range from 13,237 to 25,440. Subsequently, 12 datasets, which included the TCGA-LUAD and 11 GEO datasets, were consolidated to create 'Dataset_All'. This comprehensive dataset, 'Dataset_All', encompasses a total of 2,154 LUAD samples and 9722 genes.

**Table 1:** Clinical details of datasets.

Datasets	Sample number	Gender	Age (mean value years)	T stage	N stage	M stage	Survival status	Survival time (mean value years)	Reference
GSE3141	58	Not available	Not available	Not available	Not available	Not available	Alive (n = 26); Dead (n = 32)	2.6	^34^
GSE13213	117	Female (n = 57); Male (n = 60)	61	T1 (n = 54); T2 (n = 50); T3 (n = 8); T4 (n = 5)	N0 (n = 87); N1 (n = 8); N2 (n = 22)	M0 (n = 117)	Alive (n = 68); Dead (n = 49)	5.3	^35^
GSE14814	71	Female (n = 34); Male (n = 37)	59	Not available	Not available	Not available	Alive (n = 36); Dead (n = 35)	4.6	^36^
GSE26939	115	Female (n = 50); Male (n = 49)	64	Not available	Not available	Not available	Alive (n = 49); Dead (n = 66)	3.4	^37^
GSE30219	85	Female (n = 19); Male (n = 66)	61	T1 (n = 71); T2 (n = 12); T3 (n = 2)	N0 (n = 82); N1 (n = 3)	M0 (n = 85)	Alive (n = 40); Dead (n = 45)	6.5	^38^
GSE31210	226	Female (n = 121); Male (n = 105)	60	Not available	Not available	Not available	Alive (n = 191); Dead (n = 35)	4.7	^39^
GSE37745	106	Female (n = 60); Male (n = 46)	63	Not available	Not available	Not available	Alive (n = 29); Dead (n = 77)	5.1	^40^
GSE41271	182	Female (n = 90); Male (n = 92)	64	Not available	Not available	Not available	Alive (n = 112); Dead (n = 70)	3.8	^41^
GSE42127	133	Female (n = 65); Male (n = 68)	66	Not available	Not available	Not available	Alive (n = 90); Dead (n = 43)	4.1	^42^
GSE50081	127	Female (n = 62); Male (n = 65)	69	T1 (n = 43); T2 (n = 82); T3 (n = 2)	N0 (n = 94); N1 (n = 33)	M0 (n = 127)	Alive (n = 76); Dead (n = 51)	4.0	^43^
GSE68465	442	Female (n = 219); Male (n = 223)	64	T1 (n = 150); T2 (n = 251); T3 (n = 28) T4 (n = 11)	N0 (n = 299); N1 (n = 87); N2 (n = 53)	Not available	Alive (n = 206); Dead (n = 236)	4.4	^44^
TCGA-LUAD	492	Female (n = 266); Male (n = 226)	65	T1 (n = 167); T2 (n = 260); T3 (n = 43) T4 (n = 19)	N0 (n = 316); N1 (n = 92); N2 (n = 70) N3 (n = 2);	M0 (n = 325); M1 (n = 25)	Alive (n = 370); Dead (n = 122)	1.5	^45^

### Identification and verification of tumor subtypes

In our study, we initially sourced 1000 genes each associated with "oxidative stress" and "immunotherapy" based on their top relevance scores from GeneCards, totaling 2000 OSIRGs genes. From this pool, we eliminated duplicate genes and those not present in Dataset_All, leaving us with expression values for 1311 distinct genes. Then, we divided the entire Dataset_All into two equal parts. This distribution was carried out randomly, resulting in the 'Dataset_Training' and 'Dataset_Testing' subsets, with each accounting for 50% of the overall data. Both Dataset_Training and Dataset_Testing contained 1077 LUAD samples, each accompanied by RNA-seq data and related clinical details. Finally, the expression matrix for the 1311 genes was subjected to Consensus Clustering (CC) analysis.

In the Dataset_Training, the heatmap of the consensus matrices for k = 2 (Fig. 2C) distinctly highlighted two clusters. This distinction emphasizes that intra-cluster samples exhibit a robust correlation, while inter-cluster samples have minimal correlation. Furthermore, the overall survival (OS) prognosis showed a marked difference in two subtypes (Fig. 2D). This dataset's samples were bifurcated into Subtype_O and Subtype_M. Notably, patients classified under Subtype_M demonstrated a more favorable prognosis in contrast to those in Subtype_O. Using the same methodology, the CC-based cluster analysis was extended to Dataset_Testing, which, like its counterpart, consisted of 1077 LUAD samples with relevant expression data and survival statistics. This dataset's samples were bifurcated into Subtype_H and Subtype_P (Fig. 2E). A comparative analysis of the overall survival (OS) curves (Fig. 2F) found that patients within Subtype_H exhibited a better prognosis relative to those in Subtype_P.

### DEG and GSEA analysis

In the training dataset, we calculated the log2FC and the adjusted p-values of genes between Subtype_O and Subtype_M samples by DEG analysis. The following the GSEA results showed that Subtype_O samples are enriched in several pathways involved in cell proliferation, cycle, and metabolism (Table 2). These pathways cover a spectrum of cellular processes from energy metabolism (like glycolysis and oxidative phosphorylation) to cell growth (MYC and mTORC1 signaling) and genome maintenance (DNA repair and G2M checkpoint). Their dysregulation can significantly influence the onset and progression of various cancers. Oxidative phosphorylation is a crucial process for producing ATP, and its inherent electron transfer activities can also lead to ROS formation. On the other hand, immune pathways were found to be enriched in Subtype_M samples (Table 2). These pathways encompass critical aspects of cellular function, like immune responses, including the IL-2 STAT5 and Interferon Gamma pathways. Many, such as TNFA Signaling via NFKB and Inflammatory Response, play roles in inflammation, which can influence cancer progression. Based on these results, we labeled the Subtype_O as OX^+^ (oxidative stress) subtype, and the Subtype_M as IM^+^ (immune) subtype.

**Table 2:** Enrichment scores of significant hallmark pathways between Subtype_O and Subtype_M samples in the training dataset.

Pathway	Adjusted p-value	NES
HALLMARK_MYC_TARGETS_V1	0.02	3.69
HALLMARK_E2F_TARGETS	0.02	3.65
HALLMARK_OXIDATIVE_PHOSPHORYLATION	0.02	3.32
HALLMARK_G2M_CHECKPOINT	0.02	3.31
HALLMARK_MYC_TARGETS_V2	0.01	2.89
HALLMARK_MTORC1_SIGNALING	0.03	2.61
HALLMARK_DNA_REPAIR	0.02	2.59
HALLMARK_GLYCOLYSIS	0.02	2.31
HALLMARK_UNFOLDED_PROTEIN_RESPONSE	0.02	2.17
HALLMARK_FATTY_ACID_METABOLISM	0.02	1.82
HALLMARK_IL2_STAT5_SIGNALING	< 0.01	− 2.23
HALLMARK_COAGULATION	< 0.01	− 2.23
HALLMARK_COMPLEMENT	< 0.01	− 2.36
HALLMARK_TNFA_SIGNALING_VIA_NFKB	< 0.01	− 2.47
HALLMARK_KRAS_SIGNALING_UP	< 0.01	− 2.47
HALLMARK_IL6_JAK_STAT3_SIGNALING	< 0.01	− 2.49
HALLMARK_INTERFERON_GAMMA_RESPONSE	< 0.01	− 2.57
HALLMARK_EPITHELIAL_MESENCHYMAL_TRANSITION	< 0.01	− 2.58
HALLMARK_INFLAMMATORY_RESPONSE	< 0.01	− 2.65
HALLMARK_ALLOGRAFT_REJECTION	< 0.01	− 2.81

*NES* normalized enrichment score.

Similarly, we calculated the log2FC values, p-values of genes, and GSEA results between Subtype_H and Subtype_P samples. In the pathways enriched in Subtype_P samples (Table 3), the MYC Targets pathways emphasize the MYC oncogene's influence on cell growth and proliferation. The pathways, E2F Targets, G2M Checkpoint, and mTORC1_signaling, revolve around cell cycle progression and growth signaling. Meanwhile, Oxidative Phosphorylation and Glycolysis highlight cellular energy production mechanisms. On the other hand, several immune pathways, including Interferon Alpha Response, TNFA Signaling via NFKB, and Interferon Gamma Response, were found in the pathways enriched in Subtype_H samples. Besides, Complement, Inflammatory Response, and Allograft Rejection reflect innate and adaptive immune responses. Based on these results, we labeled the Subtype_P as OX^+^ (oxidative stress) subtype, and the Subtype_H as IM^+^ (immune) subtype. The same results in GSEA analysis from the Dataset_Testing reconfirm the robustness of these two subtypes. It worth noting that IM^+^ subtype (Subtype_M in the Dataset_Training, Subtype_H in Dataset_Testing) have a better prognosis than OX^+^ subtype (Subtype_O in the Dataset_Training, Subtype_P in Dataset_Testing) samples.

**Table 3:** Enrichment scores of significant hallmark pathways between Subtype_H and Subtype_P samples in the testing dataset.

Pathway	Adjusted p-value	NES
HALLMARK_MYC_TARGETS_V1	0.01	3.78
HALLMARK_E2F_TARGETS	0.01	3.69
HALLMARK_G2M_CHECKPOINT	0.01	3.33
HALLMARK_OXIDATIVE_PHOSPHORYLATION	0.01	3.24
HALLMARK_MTORC1_SIGNALING	0.01	2.84
HALLMARK_MYC_TARGETS_V2	0.01	2.69
HALLMARK_DNA_REPAIR	0.01	2.58
HALLMARK_UNFOLDED_PROTEIN_RESPONSE	0.01	2.52
HALLMARK_GLYCOLYSIS	0.01	2.29
HALLMARK_PROTEIN_SECRETION	0.01	2.04
HALLMARK_INTERFERON_ALPHA_RESPONSE	< 0.01	− 2.26
HALLMARK_TNFA_SIGNALING_VIA_NFKB	< 0.01	− 2.33
HALLMARK_COMPLEMENT	< 0.01	− 2.33
HALLMARK_IL2_STAT5_SIGNALING	< 0.01	− 2.35
HALLMARK_KRAS_SIGNALING_UP	< 0.01	− 2.43
HALLMARK_IL6_JAK_STAT3_SIGNALING	< 0.01	− 2.44
HALLMARK_EPITHELIAL_MESENCHYMAL_TRANSITION	< 0.01	− 2.51
HALLMARK_INTERFERON_GAMMA_RESPONSE	< 0.01	− 2.62
HALLMARK_INFLAMMATORY_RESPONSE	< 0.01	− 2.74
HALLMARK_ALLOGRAFT_REJECTION	< 0.01	− 2.96

*NES* normalized enrichment score.

### Differences in oxidative stress, TME, and immune checkpoint genes between subtypes

**Figure 3:**
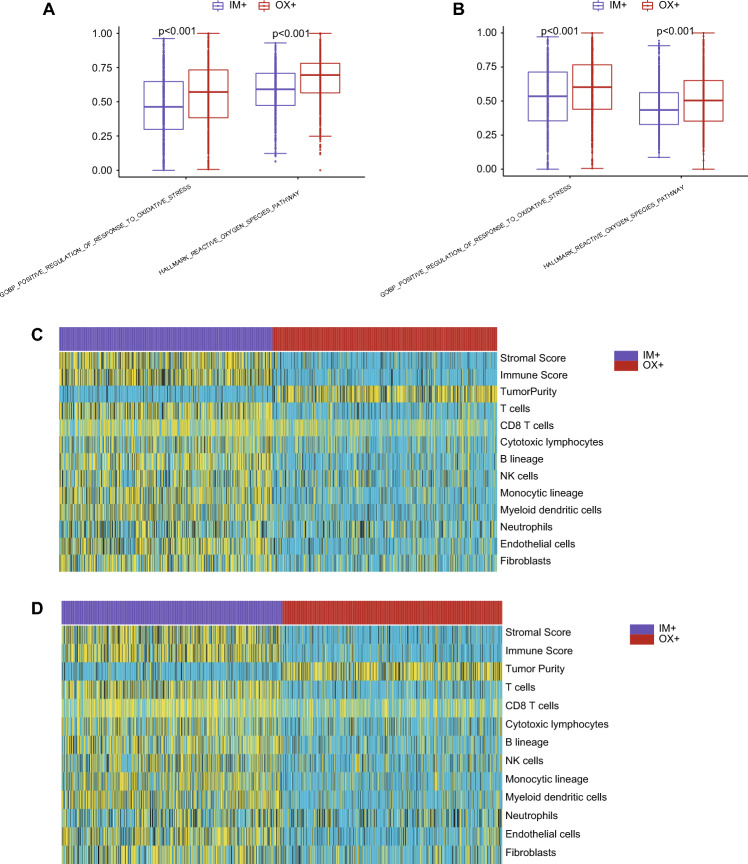
Differences of oxidative stress gene sets and TME between two LUAD subtypes. (**A**) Values of oxidative stress gene sets between two LUAD subtypes in Dataset_Training. (**B**) Values of oxidative stress gene sets between two LUAD subtypes in Dataset_Testing. (**C**) Heatmap for illustrating the values of cells from TME in Dataset_Training. The blue color indicates low value, while the yellow color indicates high value. (**D**) Heatmap for illustrating the values of cells from TME in Dataset_Testing.

We obtained gene sets related to oxidative stress from the Gene Ontology and Hallmark gene sets, and subsequently computed their enrichment scores using the GSVA package. Two gene sets related to oxidative stress, namely GOBP Positive Regulation of Response to Oxidative Stress, and Hallmark Reactive Oxygen Species Pathway, were selected. In both Dataset_Training and Dataset_Testing, OX^+^ subtype showed a significant association with activated oxidative stress, as shown in Fig. 3A,B. The distribution patterns of immune score, stroma score, tumor purity, and the proportion of immune cells were calculated for different subtypes. There was a significant difference in the distribution of these scores across subtypes. In both Dataset_Training and Dataset_Testing, the distribution of stromal score, immune score, and immune were higher in IM^+^ subtype than in OX^+^ subtype, as shown in Fig. 3C,D. However, the distribution pattern for tumor purity was the opposite, its value was higher in OX^+^ subtype than in IM^+^ subtype. Among the popular immune checkpoint genes, two of them have available expression values in Dataset_Training and Dataset_Testing. We extracted the expression levels of two immune checkpoint genes, namely PD1 (PDCD1) and CTLA-4 (CTLA4). As illustrated in Supplementary Fig. 1A–D, in most datasets, we observed significantly higher expression levels of these genes in the IM^+^ subtype compared to the OX^+^ subtype. These results reconfirmed the OX^+^ subtype is higher with oxidative stress, and IM^+^ subtype is higher with immune.

After identifying the subtype in LUAD samples, we delved into the disparities in age, gender, and TNM stages between the subtypes. It's important to mention that not all samples possess this clinical data. For instance, M stage information is only available for LUAD samples from GSE13213, GSE30219, GSE50081, and TCGA. Notably, IM^+^ subtype samples tend to be older in age (64.4 vs. 63.1, p-value < 0.01, Supplementary Fig. 2A). There's also a pronounced gender variation between the subtypes: OX^+^ subtype predominantly consists of male patients, while the IM^+^ subtype has a higher percentage of female patients (p-value < 0.01, Supplementary Fig. 2B). Regarding tumor stages, OX^+^ subtypes often have more advanced stages, such as T2/T3 (p-value < 0.01, Supplementary Fig. 2C) and N1/N2 (p-value = 0.02, Supplementary Fig. 2D), whereas IM^+^ subtypes predominantly have more localized stage tumors, like T1 and N0. For the M stage, however, there was no significant difference observed (p-value = 0.07, Supplementary Fig. 2E).

### Feature selection, model construction, and model validation

**Figure 4:**
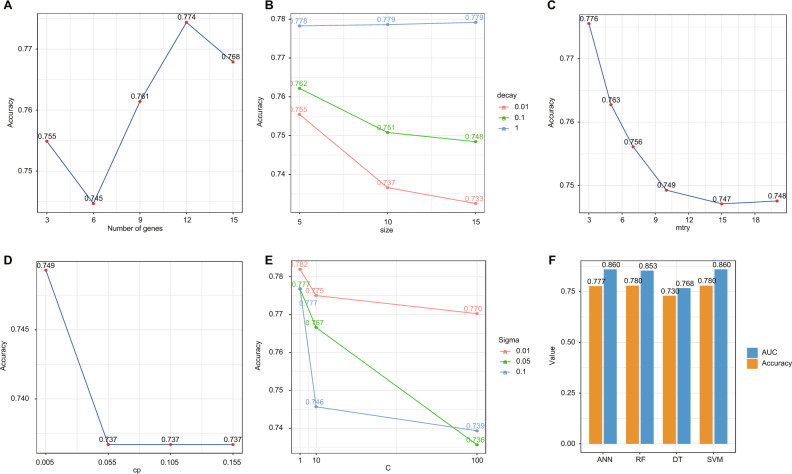
The construction and validation of machine learning models. (**A**) The selection of optimal number of genes by accuracy. The grid search of optimal parameters for ANN (**B**), RF (**C**), DT (**D**) and SVM (**E**) by accuracy values. The performance of models in the Dataset_Testing (**F**).

From the OSIRGs with available expression data in Dataset_Training, we identified 126 genes that exhibited a log2FC > 0.5 and an adjusted p-value of 0.05. The SVM-RFE approach was used to determine combinations of 3, 6, 9, 12, 15, and 18 genes. After employing fivefold cross-validation to calculate their respective accuracy values, we observed stability in the accuracy value when using 12 genes, as depicted in Fig. 4A. The 12 selected genes were: ACP1, AURKA, BIRC5, CYC1, GSTP1, HSPD1, HSPE1, MDH2, MRPL13, NDUFS1, SNRPD1, and SORD. Subsequently, we leveraged four machine learning methods—ANN, RF, DT, and SVM—for constructing models based on these 12 genes. The tuning of parameters was driven by both mean accuracy and AUC values, determined through 50 replicates of fivefold cross-validation on the Dataset_Training. Specifically: For the ANN method, the optimal parameter was identified as “size = 5” and “decay = 1”, resulting in accuracy of 0.778 (Fig. 4B) and AUC value of 0.866 (Supplementary Fig. 3A). For the RF method, the optimal parameter was identified as “mtry = 3”, resulting in accuracy of 0.776 (Fig. 4C) and AUC value of 0.856 (Supplementary Fig. 3B). For the DT method, the optimal parameter was identified as “cp = 0.005”, resulting in accuracy of 0.749 (Fig. 4D) and AUC value of 0.800 (Supplementary Fig. 3C). For SVM, the most suitable parameters were “sigma = 0.01” and “C = 1”, achieving accuracy value of 0.782 (Fig. 4E) and AUC value of 0.865 (Supplementary Fig. 3D). After setting the best parameters, the final models were constructed and then tested in the Dataset_Testing. ANN, RF, DT, and SVM achieved the AUC values of 0.860, 0.853, 0.768, and 0.860 (Fig. 4F). ANN, RF, DT, and SVM achieved the Accuracy values of 0.777, 0.780, and 0.730 and 0.780 (Fig. 4F). Among these four machine learning models, SVM showed the highest AUC and Accuracy values in Dataset_Testing. These external validation results showed that our model could accurately predict the tumor subtype of LUAD. The importance values of these 12 genes were also calculated: HSPE1 with 0.7377, CYC1 with 0.7131, HSPD1 with 0.7109, MRPL13 with 0.6929, AURKA with 0.6869, SNRPD1 with 0.6801, NDUFS1 with 0.6772, MDH2 with 0.6753, GSTP1 with 0.6735, ACP1 with 0.6734, BIRC5 with 0.6681, and SORD with 0.6679. In our SVM model, genes are assigned importance values that quantify their contribution to the classification task. A higher importance value for a particular gene indicates that it plays a more significant role in the accurate classification of LUAD subtypes. For example, HSPE1 emerged as a hub gene in our analysis because it had the highest importance value, suggesting its pivotal role in distinguishing between the OX^+^ and IM^+^ subtypes.

### The expression distribution, survival analysis and potential compounds for the hub gene

**Figure 5:**
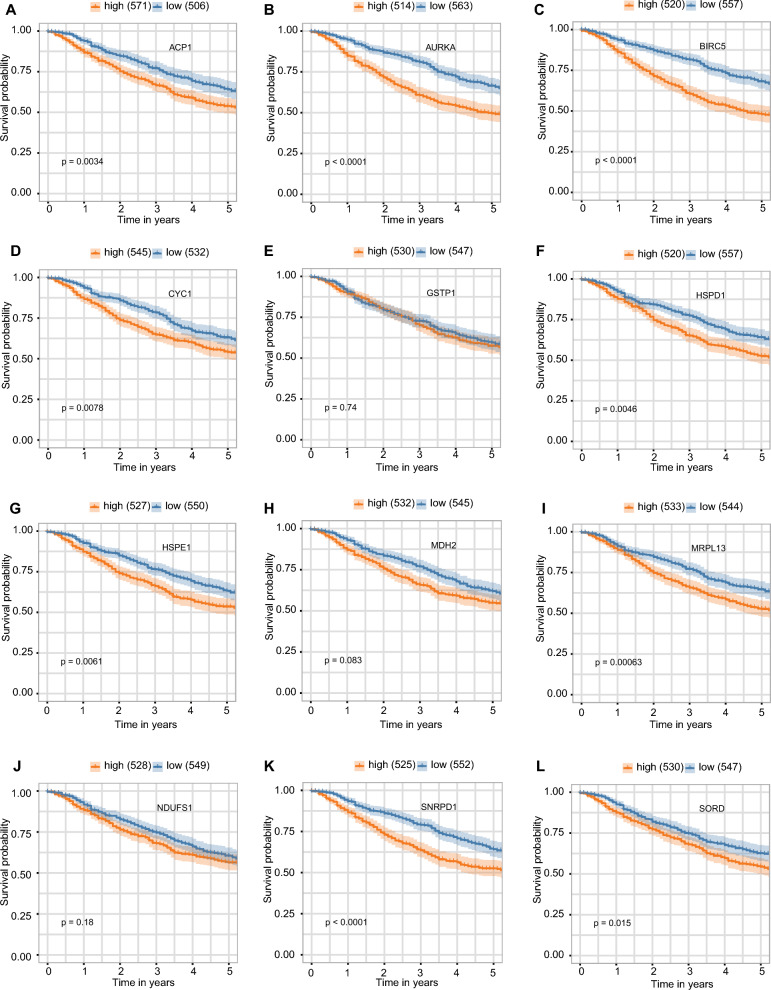
The survival analysis of 12 genes, including ACP1 (**A**), AURKA (B), BIRC5 (**C**), CYC1 (**D**), GSTP1 (**E**), HSPD1 (**F**), HSPE1 (**G**), MDH2 (**H**), MRPL13 (**I**), NDUFS1 (**J**), SNRPD1 (**K**), and SORD (**L**) in the Dataset_Training.

Then, we presented Log2FC values and adjusted p-values of 12 genes between the OX^+^ and IM^+^ subtypes in Dataset_Training and Dataset_Testing respectively (Table 4). We found that all these 12 genes were significantly higher in the OX^+^ subtype than in the IM^+^ subtype in both Dataset_Training and Dataset_Testing. It's important to emphasize that the selection of these 12 genes was based exclusively on the information derived from the Dataset_Training. The concordance result of DEGs in Dataset_Testing reconfirmed the robust elevated expression of these genes in OX^+^ subtype. Then we plotted the survival analysis of 12 genes in Dataset_Training (Fig. 5A–L) and Dataset_Testing (Supplementary Fig. 4A–L), respectively. In Dataset_Training, ACP1, AURKA, BIRC5, CYC1, HSPD1, HSPE1, MRPL13, SNRPD1, and SORD were significantly correlated with negative prognosis. In Dataset_Testing, AURKA, BIRC5, CYC1, HSPD1, HSPE1, MDH2, MRPL13, NDUFS1, SNRPD1, and SORD were significantly correlated with negative prognosis. Thus, eight genes, AURKA, BIRC5, CYC1, HSPD1, HSPE1, MRPL13, SNRPD1, and SORD were correlated with negative prognosis in both Dataset_Training and Dataset_Testing.

**Table 4:** The DEGs results of 12 genes from Dataset_Training and Dataset_Testing.

Genes	Dataset_Training log_2_FC	Dataset_Training adjusted p-value	Dataset_Testing log_2_FC	Dataset_Testing adjusted p-value
ACP1	0.788	< 0.001	0.883	< 0.001
AURKA	0.933	< 0.001	0.902	< 0.001
BIRC5	0.826	< 0.001	0.739	< 0.001
CYC1	1.023	< 0.001	0.739	< 0.001
GSTP1	0.84	< 0.001	0.631	< 0.001
HSPD1	1.054	< 0.001	1.103	< 0.001
HSPE1	1.19	< 0.001	1.024	< 0.001
MDH2	0.846	< 0.001	0.713	< 0.001
MRPL13	0.937	< 0.001	0.952	< 0.001
NDUFS1	0.862	< 0.001	0.93	< 0.001
SNRPD1	0.881	< 0.001	0.94	< 0.001
SORD	0.811	< 0.001	0.658	< 0.001

**Figure 6:**
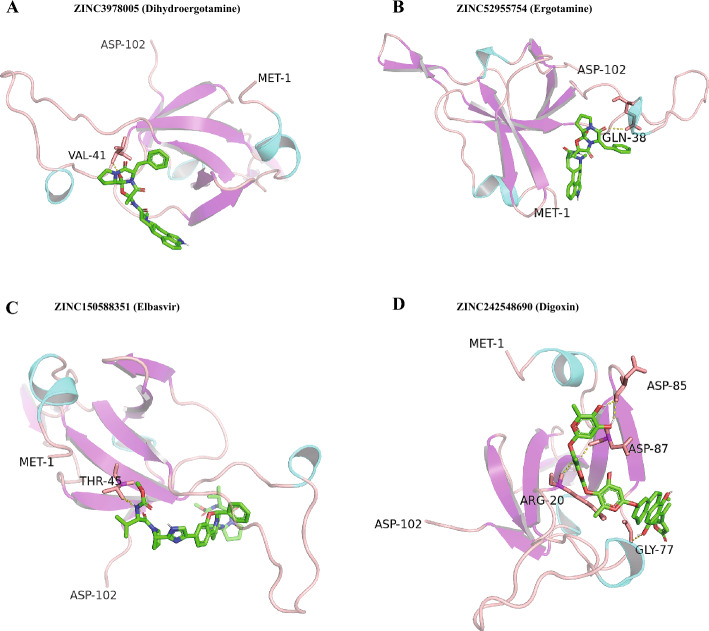
The structures of complex of (**A**) ZINC3978005 (Dihydroergotamine), (**B**) ZINC52955754 (Ergotamine), (**C**) ZINC150588351 (Elbasvir), and (**D**) ZINC242548690 (Digoxin) with HSPE1.

In order to provide potential drugs for OX^+^ subtypes, we did virtual screening analysis. HSPE1 was selected for the hub gene and the appropriate target since it had the highest importance value in the SVM model. The protein structure of HSPE1, spanning 102 residues from Met-1 to Asp-102, was sourced from Alphafold. Using this structure, we virtually screened 1295 FDA-approved compounds from the Zinc15 database using Autodock Vina. The top 10 compounds with the most favorable binding energies are detailed in Supplementary Table 2. Notably, the four compounds demonstrating the lowest binding energies were ZINC3978005 (Dihydroergotamine), ZINC52955754 (Ergotamine), ZINC150588351 (Elbasvir), and ZINC242548690 (Digoxin), with their complexes with HSPE1 visualized in Fig. 6A–D. A brief interaction analysis revealed: A hydrogen bond between ZINC3978005 and HSPE1 at Val-41. A hydrogen bond between ZINC52955754 and HSPE1 at Gln-38. A hydrogen bond between ZINC150588351 and HSPE1 at Thr-45. Four hydrogen bonds between ZINC242548690 and HSPE1 at Arg-20, Gly-77, Asp-85, and Asp-87, respectively.

## Discussion

We present a novel molecular classification technique for LUAD based on the investigation of oxidative stress and immune expression patterns. Oxidative stress has a mixed role in cancer cells as it can contribute to both the survival and death of cancer cells at different levels. Recently, ICB has shown significant improvement in the prognosis of LUAD. Molecular classification could be crucial for tailored and correct LUAD therapy. Therefore, our study aimed to discover potential subtypes among LUAD patients.

Our analysis discerned two distinct LUAD molecular subtypes. The IM^+^ subtype was marked by a profusion of immune pathways, immune cells, and immune checkpoint genes. Conversely, the OX^+^ subtype exhibited elevated oxidative stress, heightened tumor purity, and, notably, a poorer prognosis. These findings from the Dataset_Training were reconfirmed by Dataset_Testing. Immune therapies, specifically ICB that target CTLA-4 (a T-cell activation suppressor), can revitalize the immune system's prowess to decimate cancer cells, potentially prolonging patient survival^46^. While direct evidence remains elusive, the predominance of positive biomarkers like immune pathways and checkpoint genes in IM^+^ patients hints at a potential heightened responsiveness to ICB. Thus, we advocate for ICB-based treatments for the IM^+^ subtype patients. On the other hand, for the OX^+^ subtype patients, we spotlighted the protein HSPE1 – a standout in our machine learning model. Post our virtual screening process, we earmarked four compounds targeting HSPE1 as potential therapeutics for OX^+^ patients.

We constructed machine learning models leveraging four techniques: Decision Tree (DT), Support Vector Machine (SVM), Artificial Neural Networks (ANN), and Random Forest (RF). Among these, the SVM model stood out, registering the highest Accuracy and AUC values in both the Dataset_Training and Dataset_Testing. It's important to underscore that both gene selection and model construction were anchored exclusively on the Dataset_Training. An AUC value of 0.86 in the Dataset_Testing underscores the SVM's robust prediction capability when applied to a real-world, independent dataset. Furthermore, we have made our code and the trained model available on GitHub, as detailed in the Data Availability Statement. This initiative aims to foster broader utilization of our model in clinical settings.

A primary objective of our study was to pinpoint subtype-specific biomarkers and potential therapeutic agents. Through our analysis, we recognized 12 genes essential for predicting LUAD subtypes, namely: ACP1, AURKA, BIRC5, CYC1, GSTP1, HSPD1, HSPE1, MDH2, MRPL13, NDUFS1, SNRPD1, and SORD. Of these, HSPE1 stood out due to its top-tier importance in the SVM model. Consequently, our search for potential drugs pivoted towards targeting HSPE1. HSPE1 (heat shock protein family E member 1), also known as HSP10 (heat shock protein 10), encodes a protein that is part of the heat shock protein family^47^. It is intrinsically linked with HSP60, both being central to the mitochondrial chaperonin complex, assisting in mitochondrial protein folding^48^. Tumor cells are notably reliant on HSP chaperonage compared to their normal counterparts, mainly because oncoproteins in cancerous cells often misfold, necessitating amplified chaperonage activity for correction^49^. There are indications that HSPE1 might undertake diverse roles within tumor cells. For instance, its levels have been correlated with lymph node metastases^50^. Similarly, HSPE1 release has been associated with T-cell activation suppression, allowing tumors to bypass immune detection in ovarian cancers^51^. Our survival curve analysis also underscored that heightened HSPE1 levels correlate with a grim prognosis. However, the role of HSPE1 in LUAD is not clear and requires further exploration.

Oxidative stress and immunotherapy have become focal points in LUAD research. Recently, a prognostic model related to oxidative stress was provided, with the AUC of 0.660 on the 5-year survival prediction^52^. Another research proposed a nomogram with a C-index of 0.684 (95% CI, 0.656–0.712) for the recurrence-free survival of LUAD, based on clinical and oxidative stress indicators^53^. An ICB-related study tried to find the biomarker for predicting ICB efficacy^54^. Our study claims several advantages. (1) It encompasses a broader sample size, with 2,154 LUAD samples. (2) Machine learning models are harnessed, enhancing prediction accuracy. (3) We've juxtaposed both oxidative stress and immunotherapy, revealing their inverse relationship: the OX^+^ subtype exhibits diminished immune pathways, whereas IM^+^ is characterized by reduced oxidative stress pathways.

In previous research, the construction of prognostic models related to oxidative stress was often based on a limited number of datasets^52^, typically just two or three. This approach may have constrained the comprehensiveness and reliability of the findings. Incorporating a larger number of studies could significantly enhance the robustness and validity of the results. In our study, we utilized 12 distinct datasets that comprised 2,154 LUAD samples derived from varied platforms, countries, and labs. These datasets vary in sample numbers, ranging from 58 to 492. A significant challenge in integrating these datasets is the presence of batch effects. To address the batch effect, we introduced a binary transformation technique. For example, considering a specific GEO dataset where the expression of the CD8A gene fluctuates between 0 and 1000 and has a median of 500, we transformed its expression in every LUAD sample into either 0 or 1. This categorization depends on whether the expression is above or below the median value of 500. This approach was consistently applied across all genes and datasets. Our motivation behind this method stems from its straightforwardness and efficacy. This is evidenced by the PCA plot (Fig. 2B) which demonstrated that batch effects were effectively mitigated through this binary transformation. However, a potential limitation arises from the inability to apply binary transformation when only one sample contains expression data. In most clinical settings, this limitation is often overcome given the multiple LUAD patients typically present in hospitals. As a result, the initial step usually involves collecting a sufficient number of LUAD samples. In our study, GSE3141 has the lowest sample number with 58 LUAD samples. These samples allow for the effective application of binary transformation and machine learning models to their expression data. Thus, it is recommended to collect at least 58 LUAD samples before the application of binary transformation and machine learning models.

It is important to acknowledge the limitations of this study. First and foremost, the study solely relied on publicly available expression data and clinical information, and therefore, it is important to validate our findings with data from original research. Additionally, the biological roles, functions, and potential mechanisms of the identified hub genes require further investigation through experimental studies. By conducting such research, we can gain a deeper understanding of the biological processes underlying the observed associations, which may ultimately inform the development of new targeted therapies and improve clinical outcomes for patients with lung cancer.

## Conclusions

Our study aimed to create a molecular classification for lung adenocarcinoma (LUAD) by analyzing gene expression data associated with oxidative stress and immunotherapy across multiple datasets. Through our analysis, we observed significant differences in the levels of oxidative stress, prognostic features, and immune cells between the two subtypes we identified. Moreover, we identified potential targets and compounds that could enhance the survival rate of LUAD patients. Our findings provide important insights to guide the treatment of LUAD, offering a valuable reference for clinicians and researchers alike.

### Supplementary Information

Supplementary Figures.

Supplementary Tables.

## Data Availability

The code for data download and analysis can be found at https://github.com/bioCancerhznu/LUADox/.
