# Supplementary material for: Investigating subtypes of lung adenocarcinoma by oxidative stress and immunotherapy related genes
Source: Sci Rep. 2023 Nov 27;13:20930. doi: 10.1038/s41598-023-47659-8 (PMC10684862; doi:10.1038/s41598-023-47659-8)
Supplement: Supplementary file 1 — Supplementary Figures. [file 41598_2023_47659_MOESM1_ESM.pdf]

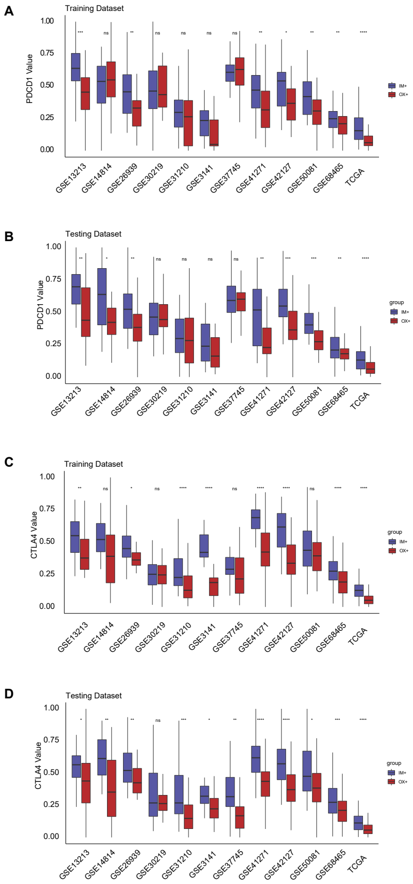

Supplementary Figure 1. Comparison of immune checkpoint gene expression levels between two LUAD subtypes.
(A) Boxplots showing the expression levels of PDCD1 between two LUAD subtypes in the Dataset_Training.
(B) Boxplots showing the expression levels of PDCD1 between two LUAD subtypes in the Dataset_Testing.
(C) Boxplots showing the expression levels of CTLA4 between two LUAD subtypes in the Dataset_Training.
(D) Boxplots showing the expression levels of CTLA4 between two LUAD subtypes in the Dataset_Testing.
*: p<0.05， **: p<0.01， ***: p<0.001, ****: p<0.001, ns: not significant.

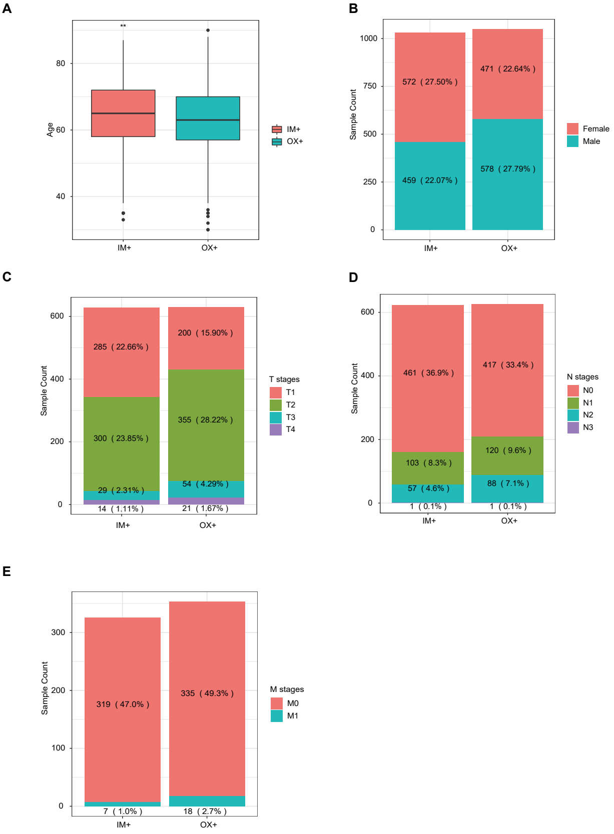

Supplementary Figure 2. The differences in age (A), gender (B), and TNM stages (C, D, E) between the subtypes. Age: T-test; Gender: chi-squared test; TNM stages: chi-squared test.

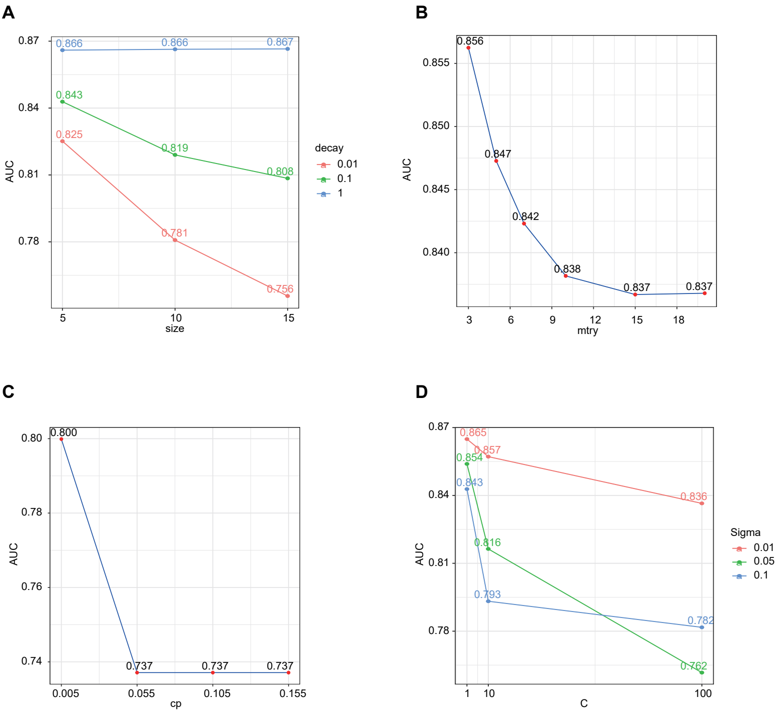

Supplementary Figure 3. The grid search of optimal parameters for ANN (A), RF (B), DT (C) and SVM (D) by AUC values.

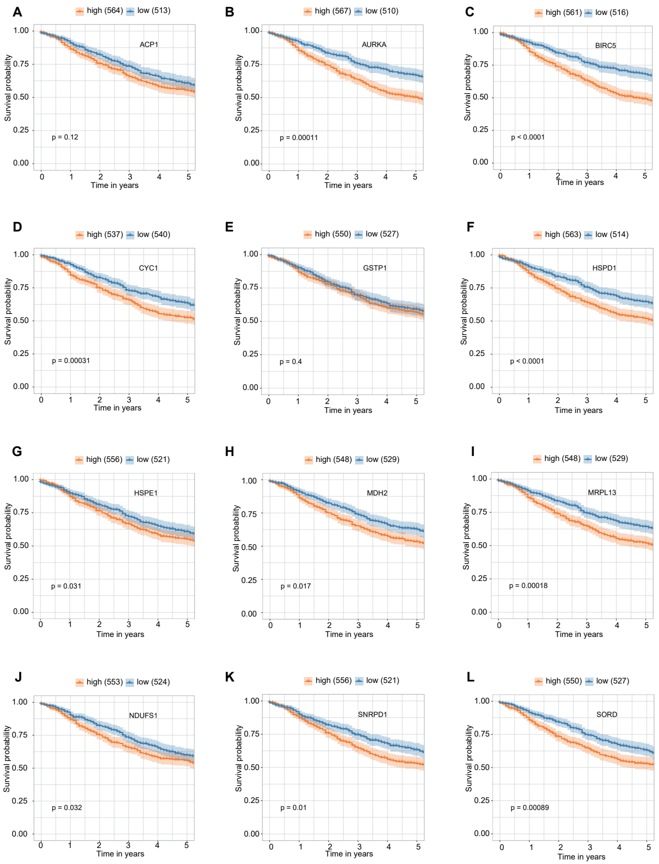

Supplementary Figure 4. The survival analysis of 12 genes, including ACP1 (A), AURKA (B), BIRC5 (C), CYC1 (D), GSTP1 (E), HSPD1 (F), HSPE1 (G), MDH2 (H), MRPL13 (I), NDUFS1 (J), SNRPD1 (K), and SORD (L) in the Dataset_Testing.
