# Supplementary material for: Investigating subtypes of lung adenocarcinoma by oxidative stress and immunotherapy related genes
Source: Sci Rep. 2023 Nov 27;13:20930. doi: 10.1038/s41598-023-47659-8 (PMC10684862; doi:10.1038/s41598-023-47659-8)
Supplement: Supplementary file 2 — Supplementary Tables. [file 41598_2023_47659_MOESM2_ESM.docx]

| **Search strategy** | **Datasets** |
| --- | --- |
| ((("lung"[MeSH Terms] OR Lung[All Fields]) AND (((((((("neoplasms"[MeSH Terms] OR Cancer[All Fields]) OR ("neoplasms"[MeSH Terms] OR Cancers[All Fields])) OR ("neoplasms"[MeSH Terms] OR Tumor[All Fields])) OR ("neoplasms"[MeSH Terms] OR Tumors[All Fields])) OR ("adenocarcinoma"[MeSH Terms] OR Adenocarcinoma[All Fields])) OR ("adenocarcinoma"[MeSH Terms] OR Adenocarcinomas[All Fields])) OR ("neoplasms"[MeSH Terms] OR Neoplasm[All Fields])) OR ("neoplasms"[MeSH Terms] OR Neoplasms[All Fields]))) AND (((("prognosis"[MeSH Terms] OR Prognosis[All Fields]) OR ("mortality"[Subheading] OR "survival"[MeSH Terms] OR Survival[All Fields])) OR Prognostic[All Fields]) OR Outcome[All Fields])) AND "Homo sapiens"[porgn] AND ("gse"[Filter] AND ("50"[n_samples] : "1000"[n_samples])) | 269 GEO datasets. |

**Supplementary Table 1.** A comprehensive search using specific keywords on the GEO database.

| **Compound name** | **Binding energy** |
| --- | --- |
| ZINC3978005 | -8.6 |
| ZINC242548690 | -8.5 |
| ZINC52955754 | -8.3 |
| ZINC150588351 | -8 |
| ZINC12503187 | -7.9 |
| ZINC150338755 | -7.9 |
| ZINC94566092 | -7.8 |
| ZINC164760756 | -7.8 |
| ZINC164760874 | -7.8 |
| ZINC6716957 | -7.7 |

**Supplementary Table 2.** The top 10 compounds with the lowest binding energies.
